# Welcome to Volume 8 of *Lung Cancer Management*

**DOI:** 10.2217/lmt-2018-0015

**Published:** 2018-12-20

**Authors:** Jennifer Straiton

**Affiliations:** 1 Future Science Group, Unitec House, 2 Albert Place, London, N3 1QB, UK

As the new Editor for *Lung Cancer Management*, it is with great excitement that I introduce the first issue of Volume 8. 2019 has lots of interesting things to be excited for; new projects, new ideas and, of course, more excellent content from all of our authors. However, before we start this new period I would like to look back over the past year and our 2018 highlights.

2018 was a year of change for the editorial team at *Lung Cancer Management* with new faces starting and the journal growing. One of the biggest changes is yet to come with our acceptance to PubMed Central; currently in the final stages of the process, we hope to have all content from the past 2 years and onwards available to search through PubMed in the near future. This is a huge step and can only be beneficial in increasing the reach of the articles we publish.

## Content highlights of 2018

The standard of content that we publish has remained high. This is due to both our authors and our reviewers who utilize our peer review and revisions policy to ensure all work is at its best.

The most-read article of 2018 also happens to be our most-read article ever published. The research team from Allegheny Health Network Cancer Institute (PA, USA) discusses the use of stereotactic body radiotherapy for early stage lung cancer and outcomes following its use in biopsy-proven and empirically treated lesions [1]. Watch this space as the authors have written not one but two follow-up articles to this piece, hopefully each proving as successful as the last.

Another 2018 article hitting our most read list is from our Editorial Board member Antonio Rossi and colleague Lucia Anna Muscarella (Casa Sollievo della Sofferenza Hospital, Italy), commenting on the presence of the *NRG1* gene in cases of non-small-cell lung cancer [2].

Looking at the use of imaging methods for staging NSCLC, Vasiliki-Konstantina I Gkogkozotou and the team from the University of Athens (Athens, Greece) investigate the use of PET/CT and brain MRI in detecting the different stages of the cancer in patients, concluding that a combination of the imaging techniques can increase accuracy in staging and may help in reducing avoidable thoracotomies and lower morbidity rates [3].

## Editorial board

Unfortunately, 2018 saw the loss of our long-term editor-in-chief, Dr David Sugarbaker, who passed away last August. The legacy he leaves behind is great and he will long be remembered as a pioneer in the treatment of mesothelioma. He completed the first lung transplant in the state of Massachusetts, developed innovative multimodality therapies and pioneered the technique of extrapleural pneumonectomy. His work will continue through the mesothelioma program he started during his time at Baylor College of Medicine, serving patients and families from around the world. Our sympathies go out to his family and colleagues; he will be sorely missed.

To the rest of our Editorial Board, we thank them for their continued input, be it in an ambassadorial, advisory or authorship role, and we look forward to working together in the coming year.

## Article outreach

To help further spread the work we publish, we continue to utilize the power of social media and share new work through platforms such as Twitter to ensure articles can be shared with the largest possible audience; if you do not already, we welcome you to follow us on Twitter (@fsglmt). In using social media it is our aim to reach all relevant stakeholders in the field of lung cancer management – including researchers, clinicians, charities, patients, academics/educators and patient advocates, just to mention a few.

Our partnership with the site Oncology Central [4] continues, giving authors the opportunity to put their work on the Oncology Central website and be seen by its wide readership base. Registration to Oncology Central is free and allows you to keep up to date with the latest developments in cancer research via unparalleled free access to the latest news, opinion, peer-reviewed journal articles, multimedia and exclusive content.

## Conclusion

Our readers remain central to the success of our journal and we welcome any feedback. Please do not hesitate to contact us with any suggestions for what you would like to see featured or any article proposals of your own. We welcome a wide range of unsolicited article proposals so please do get in touch for more information.

I would like to finish by once again thanking all of the authors and reviewers who helped to make Volume 7 possible; we hope to continue to build on this success and look forward to another great year.
